# Synthesis, crystal structure and Hirshfeld surface analysis of (*E*)-5-meth­oxy-2-[(*p*-tolyl­imino)­meth­yl]phenol

**DOI:** 10.1107/S205698902601056X

**Published:** 2026-10-20

**Authors:** G.M. Pradeepalakshmi, Valarmathy Govindaraj, Arumugaperumal Reshmadevi, Subramanian Ramasamy, Elias Jesu Packiam

**Affiliations:** a PG and Research Department of Chemistry, Seethalakshmi Ramaswami College, (Affiliated to Bharathidasan University), Tiruchirappalli-620002, Tamil Nadu, India; b Division of Chemistry School of Sciences Faculty of Engineering and Technology SRM Institute of Science and Technology, Tiruchirappalli-621 105 Tamil Nadu India; c Department of Chemistry, Indian Institute of Technology, Chennai-620 036, Tamil Nadu, India; University of Neuchâtel, Switzerland

**Keywords:** crystal structure, Schiff base, imine, Hirshfeld surface, hydrogen bond, crystal packing

## Abstract

Single-crystal X-ray diffraction has been used to elucidate the mol­ecular and crystal structures of (*E*)-5-meth­oxy-2-[(*p*-tolyl­imino)­meth­yl]phenol {systematic name: (*E*)-5-meth­oxy-2-[*N*-(4-methyl­phen­yl)carboximido­yl]phenol}, C_15_H_15_NO_2_. The Schiff base crystallizes in the ortho­rhom­bic space group *Pca*2_1_ and the mol­ecular structure is stabilized by an intra­molecular O—H⋯N hydrogen bond, with an O⋯N separation of 2.605 (4) Å and an O—H⋯N angle of 146°. The crystal packing was examined using Hirshfeld surface analysis, which reveals dominant H⋯H contacts make the largest contribution to the inter­molecular contacts (51.4%) of the surface, followed by H⋯C/C⋯H (30.9%), H⋯O/O⋯H (12.0%) and H⋯N/N⋯H (2.5%) contacts. The combined crystallographic and Hirshfeld surface analyses provide insight into the mol­ecular organization and inter­molecular forces governing the solid-state structure.

## Abstract 2

The crystal structure of the Schiff base compound (*E*)-5-meth­oxy-2-[(*p*-tolyl­imino)­meth­yl]phenol is described, highlighting its non-planar mol­ecular conformation and intra­molecular O—H⋯N hydrogen bond. Hirshfeld surface and energy-framework analyses reveal that H⋯H contacts dominate the crystal packing, with dispersion inter­actions contributing significantly to the stabilization of the mol­ecular pairs.

## 1. Chemical context

Schiff bases derived from salicyl­aldehyde are an important class of compounds owing to their structural diversity and applications in coordination chemistry, mol­ecular recognition and materials chemistry. The azomethine group together with the appropriately positioned phenolic hydroxyl group provides a useful mol­ecular scaffold for structural modification and metal coordination (Govindaraj & Ramanathan, 2014 ▸). This class of compounds has also attracted attention because of the reported biological potential of Schiff base derivatives, including anti­microbial and anti­oxidant properties (Govindaraj V *et al.*, 2026*c* ▸).
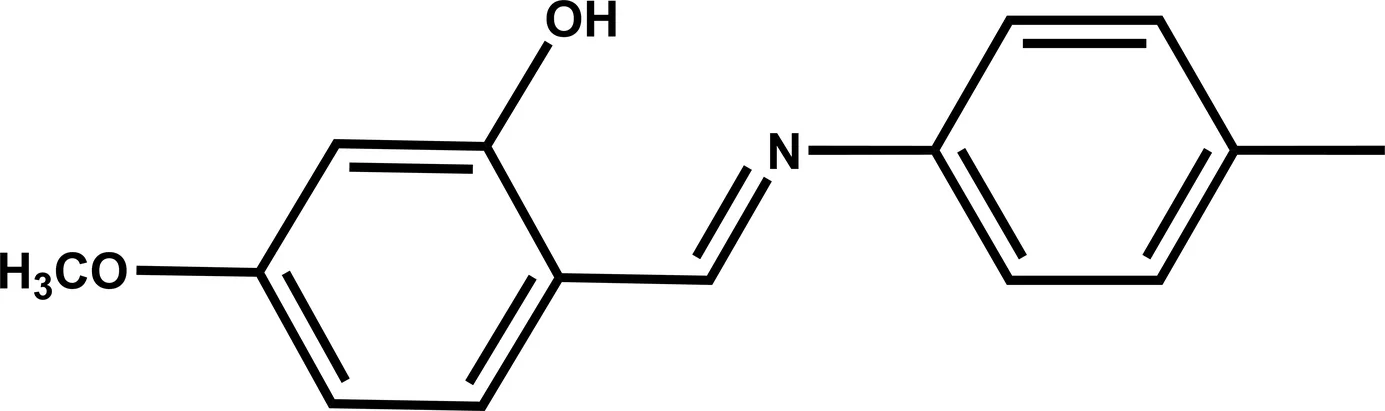


In the present work, (*E*)-5-meth­oxy-2-[(*p*-tolyl­imino)­meth­yl]phenol was synthesized by condensation of 2-hy­droxy-4-meth­oxy­benzaldehyde with *p*-toluidine. The *p*-methyl substituent was introduced to provide an electron-donating group while minimizing steric effects near the azomethine linkage. The mol­ecular and crystal structures were determined by single-crystal X-ray diffraction. Hirshfeld surface analysis and energy-framework calculations were subsequently employed to characterize the inter­molecular contacts and to assess their contributions to the consolidation of the crystal structure.

## 2. Structural commentary

**Figure 1:**
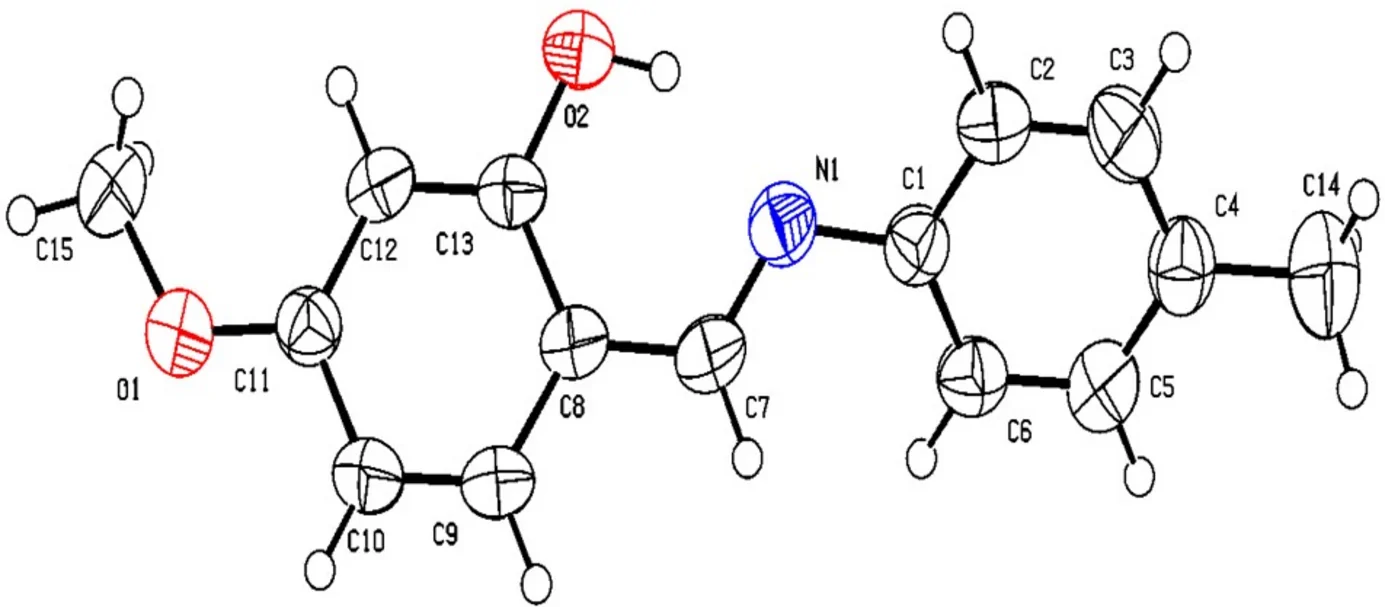
The mol­ecular structure of the title compound, showing the atom-numbering scheme and displacement ellipsoids drawn at the 50% probability level.

The mol­ecular structure of the title compound, together with the atom-numbering scheme, is shown in Fig. 1 ▸. The mol­ecule consists of two benzene rings connected through an azomethine (—CH=N—) linkage. Related Schiff-base structures have been characterized by single-crystal X-ray diffraction (Govindaraj *et al.*, 2026*a* ▸,*b* ▸). The C1–C6 ring corresponds to the *p*-tolyl ring, whereas the C8–C13 ring bears the phenolic hydroxyl and meth­oxy substituents. The two benzene ring mean planes are inclined at a dihedral angle 49.46 (16)°, indicating a non-planar mol­ecular conformation. The azomethine group adopts a *trans* configuration, as indicated by the C8—C7—N1—C1 torsion angle of −172.9 (3)°. The torsion angles C2—C1—N1—C7 [−144.2 (3)°] and C6—C1—N1—C7 [41.6 (5)°] further describe the orientation of the *p*-tolyl ring relative to the imine group. The principal bond lengths are consistent with the expected geometry of the Schiff-base framework. In particular, the N1=C7 bond is 1.284 (4) Å, while the C1—N1 and C7—C8 bonds are 1.420 (4) and 1.432 (5) Å, respectively. The phenolic C13—O2 bond length is 1.345 (4) Å, and the meth­oxy group is characterized by C11—O1 = 1.358 (4) Å and O-1–C15 = 1.406 (5) Å.

## 3. Supra­molecular features

**Figure 2:**
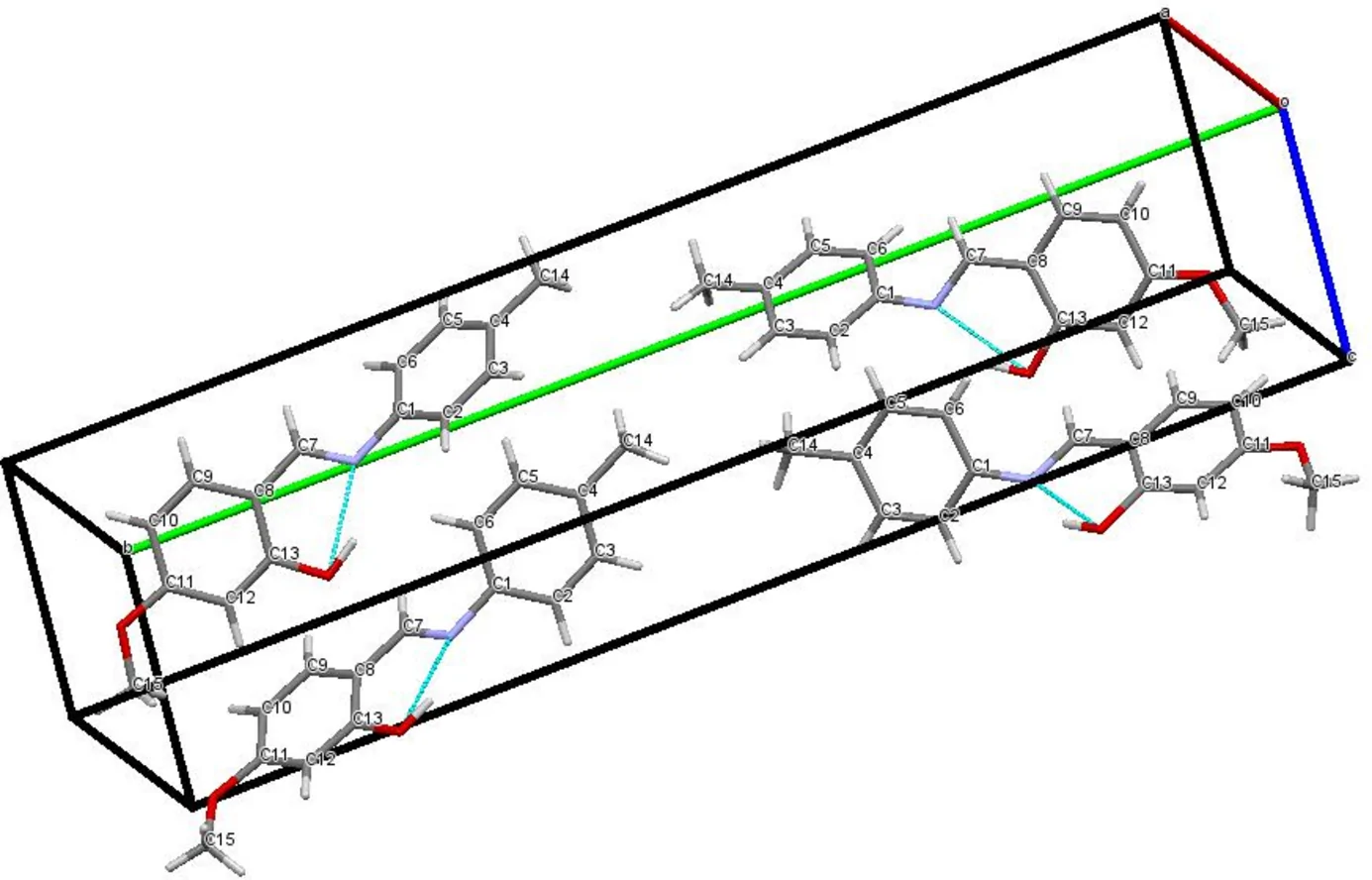
Crystal packing of the title compound, showing the arrangement of mol­ecules in the crystal viewed along the *a* axis.

The phenolic hydroxyl group forms an intra­molecular O2—H2*A*⋯N1 hydrogen bond, with an O2⋯N1 distance of 2.605 (4) Å and an O2—H2*A*⋯N1 angle of 146° (Table 1 ▸). This inter­action generates a six-membered pseudo-ring and contributes to the stabilization of the mol­ecular conformation. The crystal packing, shown in Fig. 2 ▸, is governed mainly by weak inter­molecular contacts between neighboring mol­ecules.

**Figure 3:**
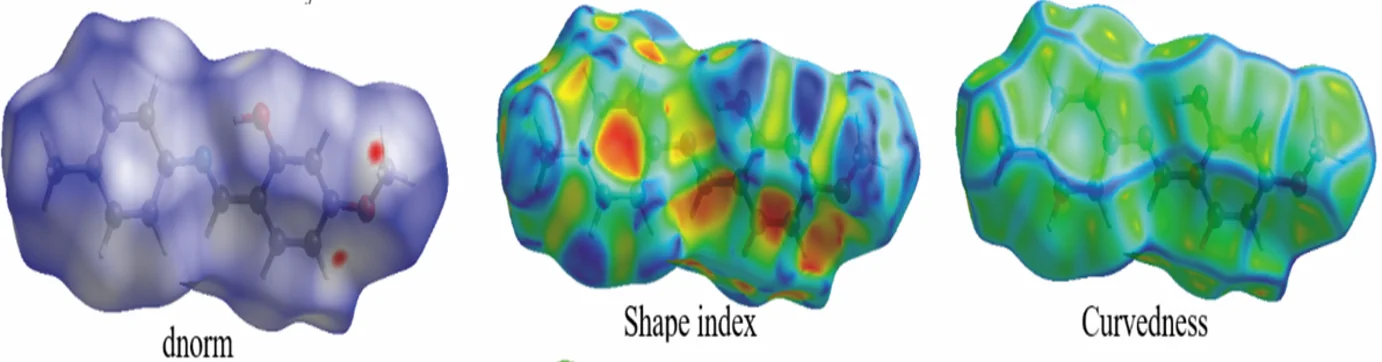
Hirshfeld surfaces of the title compound mapped over *d*_norm_, shape-index and curvedness.

**Figure 4:**
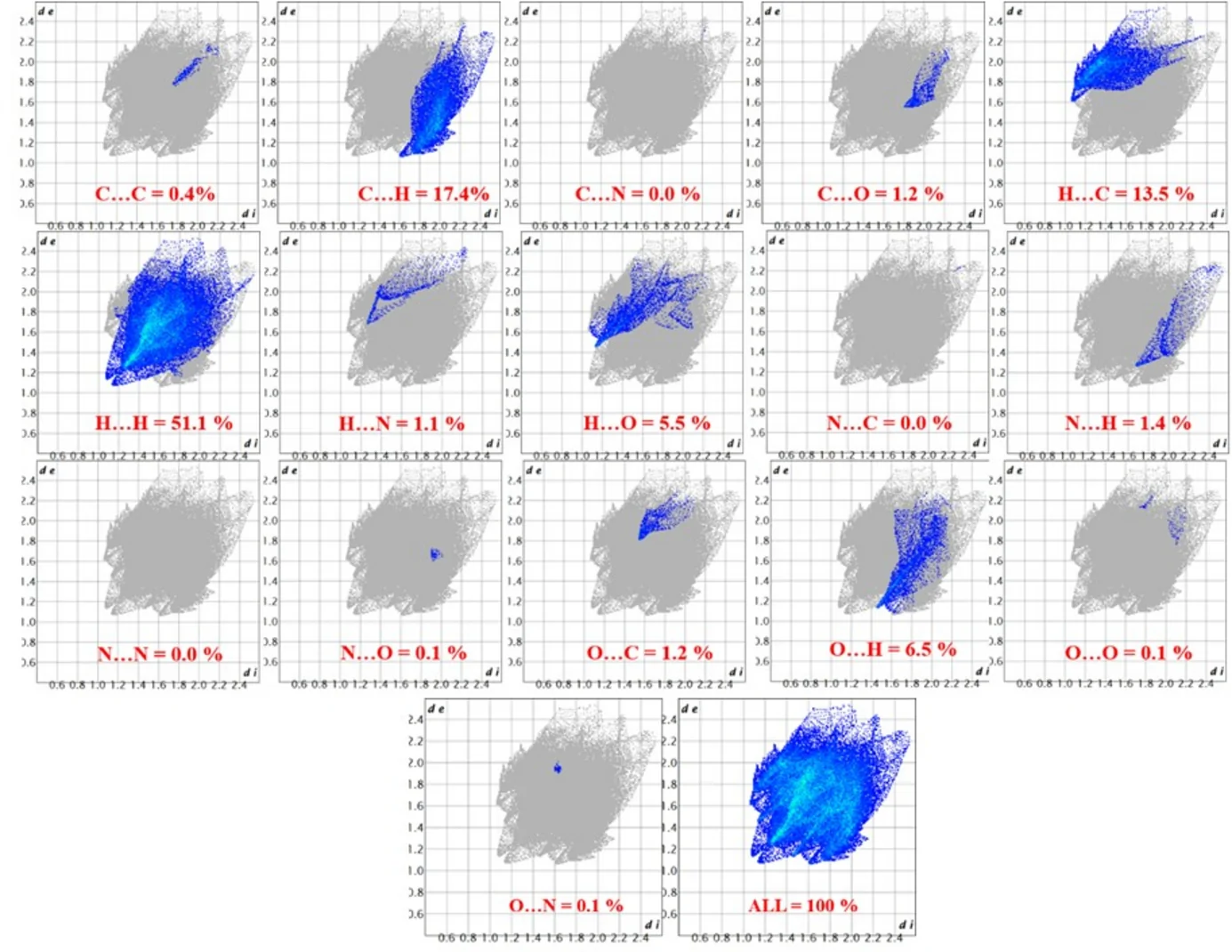
Two-dimensional fingerprint plots showing the contributions of the inter­molecular contacts in the crystal structure of the title compound.

Hirshfeld surface analysis was used to qu­antify the inter­molecular contacts (Dey *et al.*, 2024 ▸; Spackman & Jayatilaka, 2009 ▸). The Hirshfeld surface has a volume of 311.57 Å^3^ and a surface area of 299.40 Å^3^. The surface mappings are shown in Fig. 3 ▸. The two-dimensional fingerprint plots (Fig. 4 ▸) show that H⋯H contacts make the largest contribution 51.4%), followed by H⋯C/C⋯H contacts (30.9%). The H⋯O/O⋯H contacts contribute 12.0%, while H⋯N/N⋯H contacts contribute 2.5%. Smaller contributions arise from C⋯O/O⋯C (2.4%), C⋯C (0.4%) and N⋯O/O⋯N (0.2%) contacts.

## 4. Database survey

A search of the Cambridge Structural Database (CSD, version 6.01, July 2026 update; Groom *et al.*, 2016 ▸) identified the closely related Schiff-base compound (*E*)-2-[(*p*-tolyl­imino)­meth­yl]phenol (refcode CULPOO01; Nieger, 2012 ▸). Both compounds exhibit a *trans* configuration of the azomethine group, with C6—C7—N8—C9 and C8—C7—N1—C1 torsion angles of 179.41 (11) and −172.9 (3)°, respectively. The aromatic rings of the reference compound are nearly coplanar, as indicated by the C7—N8—C9—C10 torsion angle of 172.26 (12)°, whereas the title compound exhibits a more twisted arrangement, with a C2—C1—N1—C7 torsion angle of −144.2 (3)°. Both structures retain an intra­molecular O–H⋯N hydrogen bond, with donor–acceptor distances of 2.5963 (14) and 2.605 (4) Å, respectively. The meth­oxy substituent in the title compound may influence its conformational preferences and crystal packing.

## 5. Synthesis and crystallization

The title compound was prepared according to the reported procedure of Kudernac *et al.* (2009 ▸) *p*-toluidine (0.106 g, 1 mmol) and 2-hy­droxy-4-meth­oxy­benzaldehyde (0.152 g, 1 mmol) were dissolved in 15 ml of ethanol and the reaction mixture was refluxed for 3 h. After cooling to room temperature, the resulting yellow crystals were collected by filtration and dried under vacuum to give the product in approximately 0.186 g (77%) yield. Single crystals suitable for X-ray diffraction were obtained by allowing the solution to stand at room temperature for approximately 2 days, affording clear light-yellow plate-shaped crystals.

FT–IR (KBr, cm^−1^): 3546 [ν(O—H)], 1618 [ν(C=N)], 1522 [ν(C=C)], 3021 [ν(C—H)], 2922 [ν(aliphatic C—H, CH_3_)] and 1288 [ν(C—O)]. ^1^H NMR (500 MHz, DMSO-*d*_6_, δ ppm): 6.54 (*d*, 1H), 7.28 (*d*, 1H), 7.25 (*d*, 1H), 6.56 (*d*, 1H), 8.85 (*s*, 1H), 7.51 (*d*, 1H), 6.48 (*d*, 1H), 7.23 (*s*, 1H), 3.80 (*s*, 3H), 2.32 (*s*, 3H, Ar–CH_3_) and 13.81 (*s*, 1H, OH). HRMS (CH_2_Cl_2_): *m*/*z* 242.11 [*M* + H]^+^·Elemental analysis: calculated for C_15_H_15_NO_2_: C, 74.67; H, 6.27; N, 5.81; O, 13.26%; found: C, 74.66; H, 6.25; N, 5.80; O, 13.26%.

## 6. Refinement

Details of the crystal data, data collection and refinement are given in Table 2 ▸. C-bound H atoms were placed in calculated positions and refined using a riding model, while the O-bound H atom was refined independently.

## Supplementary Material

Crystal structure: contains datablock(s) I. DOI: 10.1107/S205698902601056X/tx2117sup1.cif

Structure factors: contains datablock(s) I. DOI: 10.1107/S205698902601056X/tx2117Isup3.hkl

Supporting information file. DOI: 10.1107/S205698902601056X/tx2117Isup3.cml

CCDC reference: 2480905

Additional supporting information:  crystallographic information; 3D view; checkCIF report

## Figures and Tables

**Table 1:** Hydrogen-bond geometry (Å, °)

*D*—H⋯*A*	*D*—H	H⋯*A*	*D*⋯*A*	*D*—H⋯*A*
O2—H2*A*⋯N1	0.92	1.79	2.605 (4)	146

**Table 2:** Experimental details

Crystal data	
Chemical formula	C_15_H_15_NO_2_
*M*_r_	241.28
Crystal system, space group	Orthorhombic, *Pca*2_1_
Temperature (K)	300
*a*, *b*, *c* (Å)	7.2146 (7), 28.256 (3), 6.2413 (6)
*V* (Å^3^)	1272.3 (2)
*Z*	4
Radiation type	Mo *K*α
μ (mm^−1^)	0.08
Crystal size (mm)	0.34 × 0.22 × 0.08

Data collection	
Diffractometer	Bruker D8 QUEST
Absorption correction	Multi-scan (*SADABS*; Krause *et al.*, 2015 ▸)
*T*_min_, *T*_max_	0.972, 0.994
No. of measured, independent and observed [*I* > 2σ(*I*)] reflections	29157, 3164, 2247
*R*_int_	0.061
(sin θ/λ)_max_ (Å^−1^)	0.667

Refinement	
*R*[*F*^2^ > 2σ(*F*^2^)], *wR*(*F*^2^), *S*	0.057, 0.151, 1.06
No. of reflections	3164
No. of parameters	167
No. of restraints	1
H-atom treatment	H atoms treated by a mixture of independent and constrained refinement
Δρ_max_, Δρ_min_ (e Å^−3^)	0.19, −0.17
Absolute structure	Flack *x* determined using 786 quotients [(*I*^+^)−(*I*^−^)]/[(*I*^+^)+(*I*^−^)] (Parsons *et al.*, 2013 ▸)
Absolute structure parameter	0.2 (6)

Computer programs: *Instrument Service* and *SAINT* V8.41 (Bruker, 2016 ▸), *SHELXT2018/2* (Sheldrick, 2015*a* ▸), *SHELXL2019/2* (Sheldrick, 2015*b* ▸) and *ShelXle* (Hübschle *et al.*, 2011 ▸).

## supplementary crystallographic information

### (E)-5-Methoxy-2-[N-(4-methylphenyl)carboximidoyl]phenol Crystal data

**Table:**

C_15_H_15_NO_2_	*D*_x_ = 1.26 Mg m^−^^3^
*M**_r_* = 241.28	Mo *K*α radiation, λ = 0.71073 Å
Orthorhombic, *P**c**a*2_1_	Cell parameters from 6384 reflections
*a* = 7.2146 (7) Å	θ = 2.9–23.7°
*b* = 28.256 (3) Å	µ = 0.08 mm^−^^1^
*c* = 6.2413 (6) Å	*T* = 300 K
*V* = 1272.3 (2) Å^3^	Plate, clear light yellow
*Z* = 4	0.34 × 0.22 × 0.08 mm
*F*(000) = 512	

### (E)-5-Methoxy-2-[N-(4-methylphenyl)carboximidoyl]phenol Data collection

**Table:**

Bruker D8 QUEST diffractometer	3164 independent reflections
Radiation source: microfocus sealed tube, INCOATEC IµS 3.0	2247 reflections with *I* > 2σ(*I*)
Multilayer mirror monochromator	*R*_int_ = 0.061
Detector resolution: 7.391 pixels mm^-1^	θ_max_ = 28.3°, θ_min_ = 2.2°
ω and φ scans	*h* = −9→9
Absorption correction: multi-scan (SADABS; Krause *et al.*, 2015)	*k* = −36→37
*T*_min_ = 0.972, *T*_max_ = 0.994	*l* = −8→8
29157 measured reflections	

### (E)-5-Methoxy-2-[N-(4-methylphenyl)carboximidoyl]phenol Refinement

**Table:**

Refinement on *F*^2^	Hydrogen site location: inferred from neighbouring sites
Least-squares matrix: full	H atoms treated by a mixture of independent and constrained refinement
*R*[*F*^2^ > 2σ(*F*^2^)] = 0.057	*w* = 1/[σ^2^(*F*_o_^2^) + (0.0623*P*)^2^ + 0.323*P*] where *P* = (*F*_o_^2^ + 2*F*_c_^2^)/3
*wR*(*F*^2^) = 0.151	(Δ/σ)_max_ = 0.007
*S* = 1.06	Δρ_max_ = 0.19 e Å^−^^3^
3164 reflections	Δρ_min_ = −0.17 e Å^−^^3^
167 parameters	Absolute structure: Flack *x* determined using 786 quotients [(*I*^+^)-(*I*^-^)]/[(*I*^+^)+(*I*^-^)] (Parsons *et al.*, 2013)
1 restraint	Absolute structure parameter: 0.2 (6)
Primary atom site location: Intrinsic Phasing	

### (E)-5-Methoxy-2-[N-(4-methylphenyl)carboximidoyl]phenol Special details

**Table:**

Geometry. All esds (except the esd in the dihedral angle between two l.s. planes) are estimated using the full covariance matrix. The cell esds are taken into account individually in the estimation of esds in distances, angles and torsion angles; correlations between esds in cell parameters are only used when they are defined by crystal symmetry. An approximate (isotropic) treatment of cell esds is used for estimating esds involving l.s. planes.
Refinement. The diffraction data were processed using SAINT (Bruker, 2016) and absorption correction was applied using SADABS (Krause *et al.*, 2015). The structure was solved by intrinsic phasing using SHELXT 2018/2 and refined by full-matrix least-squares methods against *F*^2^ using SHELXL-2019/3 (Sheldrick, 2015a,b). All non-H atoms were refined anisotropically.

### (E)-5-Methoxy-2-[N-(4-methylphenyl)carboximidoyl]phenol Fractional atomic coordinates and isotropic or equivalent isotropic displacement parameters (Å^2^)

**Table:**

	*x*	*y*	*z*	*U*_iso_*/*U*_eq_	
C1	0.5091 (4)	0.31881 (12)	0.3712 (6)	0.0444 (8)	
C2	0.5694 (5)	0.35917 (12)	0.4749 (6)	0.0518 (9)	
H2	0.618437	0.356993	0.612401	0.062*	
C3	0.5574 (5)	0.40250 (13)	0.3759 (8)	0.0585 (10)	
H3	0.601158	0.429228	0.446663	0.070*	
C4	0.4811 (5)	0.40729 (12)	0.1718 (7)	0.0558 (10)	
C5	0.4216 (5)	0.36685 (12)	0.0713 (7)	0.0545 (9)	
H5	0.372322	0.368988	−0.066013	0.065*	
C6	0.4329 (4)	0.32304 (13)	0.1688 (6)	0.0491 (8)	
H6	0.389169	0.296313	0.098084	0.059*	
C7	0.5508 (4)	0.23666 (12)	0.3821 (6)	0.0463 (8)	
H7	0.590493	0.238534	0.240619	0.056*	
C8	0.5352 (4)	0.19109 (11)	0.4809 (5)	0.0433 (8)	
C9	0.5914 (4)	0.15020 (12)	0.3729 (6)	0.0474 (8)	
H9	0.639037	0.153035	0.234987	0.057*	
C10	0.5789 (4)	0.10639 (12)	0.4623 (6)	0.0481 (8)	
H10	0.622562	0.080033	0.388681	0.058*	
C11	0.5001 (4)	0.10124 (12)	0.6659 (6)	0.0445 (8)	
C12	0.4384 (5)	0.14066 (11)	0.7776 (6)	0.0462 (8)	
H12	0.383505	0.137234	0.911506	0.055*	
C13	0.4595 (4)	0.18514 (11)	0.6879 (6)	0.0441 (8)	
C14	0.4622 (7)	0.45488 (14)	0.0664 (10)	0.0826 (14)	
H14A	0.536888	0.477583	0.141530	0.124*	
H14B	0.334792	0.464653	0.070195	0.124*	
H14C	0.502843	0.452796	−0.079713	0.124*	
C15	0.4117 (7)	0.04730 (15)	0.9405 (7)	0.0764 (13)	
H15A	0.285977	0.058395	0.938838	0.115*	
H15B	0.479579	0.063565	1.050459	0.115*	
H15C	0.413186	0.013907	0.968858	0.115*	
N1	0.5117 (4)	0.27501 (10)	0.4827 (5)	0.0481 (7)	
O1	0.4944 (4)	0.05613 (9)	0.7406 (4)	0.0580 (7)	
O2	0.4075 (4)	0.22284 (9)	0.8050 (4)	0.0624 (8)	
H2A	0.428 (6)	0.2502 (14)	0.727 (5)	0.094*	

### (E)-5-Methoxy-2-[N-(4-methylphenyl)carboximidoyl]phenol Atomic displacement parameters (Å^2^)

**Table:**

	*U*^11^	*U*^22^	*U*^33^	*U*^12^	*U*^13^	*U*^23^
C1	0.0395 (16)	0.049 (2)	0.044 (2)	0.0026 (13)	0.0062 (17)	0.0003 (15)
C2	0.0496 (18)	0.061 (2)	0.0447 (19)	−0.0015 (15)	−0.0016 (17)	−0.0056 (19)
C3	0.0531 (19)	0.053 (2)	0.070 (3)	−0.0063 (16)	−0.002 (2)	−0.009 (2)
C4	0.051 (2)	0.054 (2)	0.063 (3)	−0.0020 (16)	0.004 (2)	0.0060 (19)
C5	0.0487 (19)	0.063 (2)	0.052 (2)	0.0035 (16)	0.0005 (16)	0.0035 (18)
C6	0.0488 (17)	0.055 (2)	0.044 (2)	−0.0037 (15)	−0.0008 (16)	−0.0053 (15)
C7	0.0419 (16)	0.060 (2)	0.0370 (18)	0.0005 (14)	−0.0018 (14)	−0.0006 (16)
C8	0.0412 (16)	0.0533 (19)	0.0354 (18)	0.0018 (13)	−0.0003 (15)	−0.0039 (16)
C9	0.0445 (17)	0.060 (2)	0.0376 (17)	0.0017 (15)	0.0043 (15)	−0.0076 (17)
C10	0.0480 (17)	0.0534 (19)	0.0429 (18)	0.0049 (14)	0.0013 (16)	−0.0088 (17)
C11	0.0450 (17)	0.0473 (19)	0.0412 (19)	−0.0006 (14)	−0.0057 (16)	−0.0037 (15)
C12	0.0481 (17)	0.057 (2)	0.0332 (17)	0.0024 (15)	0.0029 (15)	0.0009 (15)
C13	0.0422 (16)	0.052 (2)	0.0381 (18)	0.0042 (14)	0.0011 (15)	−0.0061 (15)
C14	0.085 (3)	0.063 (3)	0.100 (4)	0.002 (2)	−0.009 (3)	0.015 (3)
C15	0.115 (4)	0.062 (2)	0.052 (2)	0.005 (2)	0.018 (2)	0.010 (2)
N1	0.0457 (14)	0.0526 (17)	0.0461 (16)	0.0012 (12)	0.0017 (15)	0.0001 (14)
O1	0.0721 (18)	0.0509 (14)	0.0510 (15)	0.0016 (12)	0.0041 (12)	−0.0008 (12)
O2	0.0848 (19)	0.0548 (14)	0.0474 (16)	0.0107 (14)	0.0176 (14)	−0.0056 (13)

### (E)-5-Methoxy-2-[N-(4-methylphenyl)carboximidoyl]phenol Geometric parameters (Å, º)

**Table:**

C1—C2	1.381 (5)	C9—C10	1.361 (5)
C1—C6	1.383 (5)	C9—H9	0.9300
C1—N1	1.420 (4)	C10—C11	1.399 (5)
C2—C3	1.374 (5)	C10—H10	0.9300
C2—H2	0.9300	C11—O1	1.358 (4)
C3—C4	1.394 (6)	C11—C12	1.387 (4)
C3—H3	0.9300	C12—C13	1.384 (4)
C4—C5	1.372 (5)	C12—H12	0.9300
C4—C14	1.503 (5)	C13—O2	1.345 (4)
C5—C6	1.382 (5)	C14—H14A	0.9600
C5—H5	0.9300	C14—H14B	0.9600
C6—H6	0.9300	C14—H14C	0.9600
C7—N1	1.284 (4)	C15—O1	1.406 (5)
C7—C8	1.432 (5)	C15—H15A	0.9600
C7—H7	0.9300	C15—H15B	0.9600
C8—C9	1.398 (4)	C15—H15C	0.9600
C8—C13	1.412 (4)	O2—H2A	0.92 (5)
			
C2—C1—C6	118.8 (3)	C9—C10—C11	119.6 (3)
C2—C1—N1	119.0 (3)	C9—C10—H10	120.2
C6—C1—N1	121.9 (3)	C11—C10—H10	120.2
C3—C2—C1	120.3 (4)	O1—C11—C12	124.8 (3)
C3—C2—H2	119.8	O1—C11—C10	114.9 (3)
C1—C2—H2	119.8	C12—C11—C10	120.2 (3)
C2—C3—C4	121.5 (4)	C13—C12—C11	119.4 (3)
C2—C3—H3	119.3	C13—C12—H12	120.3
C4—C3—H3	119.3	C11—C12—H12	120.3
C5—C4—C3	117.4 (3)	O2—C13—C12	117.9 (3)
C5—C4—C14	121.1 (4)	O2—C13—C8	120.7 (3)
C3—C4—C14	121.5 (4)	C12—C13—C8	121.4 (3)
C4—C5—C6	121.8 (4)	C4—C14—H14A	109.5
C4—C5—H5	119.1	C4—C14—H14B	109.5
C6—C5—H5	119.1	H14A—C14—H14B	109.5
C5—C6—C1	120.2 (3)	C4—C14—H14C	109.5
C5—C6—H6	119.9	H14A—C14—H14C	109.5
C1—C6—H6	119.9	H14B—C14—H14C	109.5
N1—C7—C8	122.1 (3)	O1—C15—H15A	109.5
N1—C7—H7	119.0	O1—C15—H15B	109.5
C8—C7—H7	119.0	H15A—C15—H15B	109.5
C9—C8—C13	117.1 (3)	O1—C15—H15C	109.5
C9—C8—C7	120.8 (3)	H15A—C15—H15C	109.5
C13—C8—C7	122.1 (3)	H15B—C15—H15C	109.5
C10—C9—C8	122.3 (3)	C7—N1—C1	119.9 (3)
C10—C9—H9	118.8	C11—O1—C15	119.0 (3)
C8—C9—H9	118.8	C13—O2—H2A	109.5
			
C6—C1—C2—C3	−1.4 (5)	C9—C10—C11—O1	−179.9 (3)
N1—C1—C2—C3	−175.8 (3)	C9—C10—C11—C12	−1.3 (5)
C1—C2—C3—C4	1.4 (6)	O1—C11—C12—C13	177.1 (3)
C2—C3—C4—C5	−1.3 (6)	C10—C11—C12—C13	−1.4 (5)
C2—C3—C4—C14	178.0 (4)	C11—C12—C13—O2	−176.5 (3)
C3—C4—C5—C6	1.2 (6)	C11—C12—C13—C8	2.8 (5)
C14—C4—C5—C6	−178.0 (4)	C9—C8—C13—O2	177.9 (3)
C4—C5—C6—C1	−1.3 (5)	C7—C8—C13—O2	−3.4 (4)
C2—C1—C6—C5	1.4 (5)	C9—C8—C13—C12	−1.3 (4)
N1—C1—C6—C5	175.6 (3)	C7—C8—C13—C12	177.3 (3)
N1—C7—C8—C9	−175.3 (3)	C8—C7—N1—C1	−172.9 (3)
N1—C7—C8—C13	6.1 (5)	C2—C1—N1—C7	−144.2 (3)
C13—C8—C9—C10	−1.5 (4)	C6—C1—N1—C7	41.6 (5)
C7—C8—C9—C10	179.8 (3)	C12—C11—O1—C15	3.6 (5)
C8—C9—C10—C11	2.8 (5)	C10—C11—O1—C15	−177.8 (3)

### (E)-5-Methoxy-2-[N-(4-methylphenyl)carboximidoyl]phenol Hydrogen-bond geometry (Å, º)

**Table:**

*D*—H···*A*	*D*—H	H···*A*	*D*···*A*	*D*—H···*A*
O2—H2*A*···N1	0.92	1.79	2.605 (4)	146
